# Signaling via guanylyl cyclase C: cGMP, Src and p21

**DOI:** 10.1186/1471-2210-11-S1-P4

**Published:** 2011-08-01

**Authors:** Nirmalya Basu, Sayanti Saha, Sandhya S Visweswariah

**Affiliations:** 1 Department of Molecular Reproduction, Development and Genetics, Indian Institute of Science,Bangalore 560012, India; 2 Fox Chase Cancer Center, Philadelphia, Pa 19111, USA

## Background

Guanylyl cyclase C (GC-C) is a receptor expressed in intestinal epithelial cells and activation of GCC by its ligands elevates intracellular cGMP which results in an inhibition of cell proliferation [1]. Multiple regulatory mechanisms operate in GC-C to modulate its activity, and include ligand binding to the extracellular domain, ATP binding to the kinase homology domain and additional structural features that link the kinase homology domain to the C-terminal guanylyl cyclase domain [2]. The persistent expression of GC-C in colorectal carcinomas and occult metastases makes it a marker for malignancy.

## Results

We have investigated cross-talk of GC-C with additional signalling pathways in the intestinal cell in order to investigate its role in cell proliferation. Activation of c-src in human colonic cells results in down-regulation of cGMP production by GC-C, and through mutational analysis and *invitro* and *in vivo* assays, we show that GC-C is a substrate for phosphorylation by c-src. This phosphorylation now results in a reduction in guanylyl cyclase activity. Phosphorylation of GC-C by c-src allows the docking of the c-src SH2 domain to phosphorylated GC-C, which can now result in further activation of c-src. This feed forward mechanism of activation of c-src, induced by novel cross-talk between a receptor guanylyl cyclase and a tyrosine kinase, may have important implications in colonic tumour progression [3]. To identify other downstream effects of cGMP, we have performed microarray analysis on T84 colon carcinoma cells treated with the heat-stable enterotoxin. One of the genes significantly up-regulated, both at the levels of transcript and protein, was the cell-cycle inhibitor, p21. Treatment of T84 cells with the stable toxin peptide allowed cells to enter a cGMP-dependent senescence programme. The cells show all the hallmarks of senescent cells, including flattened cell morphology, positive staining for SA-ß Gal and formation of senescence-associated heterochromatic foci. Activation of senescence and loss of tumorigenicity in these cells is crucially dependent on the up-regulation of p21 and therefore can contribute to ST induced-cytostasis.

## Conclusion

These observations may provide an explanation for the reduced incidence of colon-carcinoma seen in countries where the incidence of ST-mediated diarrhoea is higher. Moreover, our results also highlight the fact that the production of cGMP in intestinal epithelial cells is exquisitely regulated, and cGMP, in turn may regulate a number of pathways with diverse physiological effects.
